# High Resolution Analysis of the Chromatin Landscape of the IgE Switch Region in Human B Cells

**DOI:** 10.1371/journal.pone.0024571

**Published:** 2011-09-20

**Authors:** Sandeep Dayal, Jakub Nedbal, Philip Hobson, Alison M. Cooper, Hannah J. Gould, Martin Gellert, Gary Felsenfeld, David J. Fear

**Affiliations:** 1 Laboratory of Molecular Biology, National Institute of Diabetes and Digestive and Kidney Diseases, National Institutes of Health, Bethesda, Maryland, United States of America; 2 Division of Asthma, Allergy and Lung Biology, King's College London, London, United Kingdom; 3 Medical Research Council and Asthma UK Centre in Allergic Mechanisms of Asthma, King's College London, London, United Kingdom; 4 Randall Division of Cell and Molecular Biophysics, King's College London, London, United Kingdom; Florida State University, United States of America

## Abstract

Antibodies are assembled by a highly orchestrated series of recombination events during B cell development. One of these events, class switch recombination, is required to produce the IgG, IgE and IgA antibody isotypes characteristic of a secondary immune response. The action of the enzyme activation induced cytidine deaminase is now known to be essential for the initiation of this recombination event. Previous studies have demonstrated that the immunoglobulin switch regions acquire distinct histone modifications prior to recombination. We now present a high resolution analysis of these histone modifications across the IgE switch region prior to the initiation of class switch recombination in primary human B cells and the human CL-01 B cell line. These data show that upon stimulation with IL-4 and an anti-CD40 antibody that mimics T cell help, the nucleosomes of the switch regions are highly modified on histone H3, accumulating acetylation marks and tri-methylation of lysine 4. Distinct peaks of modified histones are found across the switch region, most notably at the 5′ splice donor site of the germline (I) exon, which also accumulates AID. These data suggest that acetylation and K4 tri-methylation of histone H3 may represent marks of recombinationally active chromatin and further implicates splicing in the regulation of AID action.

## Introduction

Antibodies, which are essential components of vertebrate adaptive immunity, are produced as a result of complex genome rearrangements and mutation events in the B cell receptor loci. In developing B lymphocytes, V(D)J recombination at immunoglobulin heavy and light chain loci results in a diverse repertoire of antigen binding specificities necessary for the recognition of a spectrum of foreign antigens. During the immune response, somatic hypermutation (SHM) and affinity maturation refine these specificities through the introduction of mutations into the variable regions while class switch recombination (CSR) exchanges the constant regions of the immunoglobulin heavy chains (IgH) to produce the different antibody isotypes, or classes. The germline IgH locus consists of a linear array of constant region (C_H_) genes spanning over one hundred kilobases, with Cμ, which encodes IgM, proximal to the rearranged V(D)J gene segments [1]. With the exception of Cδ, each downstream C_H_ gene contains an individual promoter, short intervening (I) exon and a 2–10 kb switch (S) region followed by coding region exons [2]. During CSR, DNA double strand breaks (DSBs) are generated in the donor (initially Sμ) and downstream target switch regions [3], [4]. These switch regions then recombine to place the target C_H_ immediately downstream of the assembled V(D)J gene segments, allowing the expression of a new immunoglobulin isotype whilst maintaining antigen specificity [5].

In the past few decades some of the key components and mechanistic steps of CSR have been elucidated. The enzyme activation induced cytidine deaminase (AID) has been shown to play a central role in both SHM and CSR [6]–[8]. Extracellular cytokine signals initiate CSR by activating transcription of donor and target C_H_ genes (germline transcription, GLT) [9] and upon further signalling by CD40-ligand, or one of its analogs [10], [11], recombination ensues. AID deaminates deoxycytidines within IgH switch regions, converting them to deoxyuridines [12]–[14]. If there are two close-lying events on opposite strands, the resulting U:G mismatches initiate a cascade of activities that ultimately result in the formation of DNA DSBs in the donor and target switch regions [15], [16]. DNA repair mechanisms resolve these DSBs, ligating the donor and target switch regions, moving the target C_H_ exons adjacent to the expressed V(D)J gene segments [17].

Chromatin structure is known to play an important role in most, if not all, vertebrate processes that require direct access to DNA, such as transcription, replication and recombination. In many cases, distinct post-translational modifications in the N-terminal histone tails correlate strongly with “active” or “silent” transcriptional states. For example, acetylation of histones H3 and H4 marks regions of transcriptionally active chromatin, whereas tri-methylation of histone H3 at lysine 27 is associated with transcriptionally silent loci [18]. Local chromatin accessibility is changed by alterations in nucleosome positioning through ATP-dependent remodelling activities or through the recruitment of histone modification enzymes such as histone acetyl transferases (HATs) or histone methyl transferases (HMTs) [19].

Several lines of evidence have suggested that switch region chromatin structure plays a key role in promoting a permissive environment required for AID attack. Hyperacetylation of histones H3 and H4 and tri-methylation of histone H3 on lysine 4 (K4) and lysine 9 (K9) have previously been shown to be associated with activated switch regions in both mice and humans [20]–[25]. However, the present work is the first to examine chromatin structure over a human switch region at high resolution.

We have focused on the histone modifications that occur prior to CSR to IgE in human B cells. IgE is the antibody class that mediates the allergic response and its regulation is therefore of considerable interest. In particular, we have investigated the changes in chromatin structure that occur under conditions that induce ε germline gene transcription; an event that precedes, and is necessary for, CSR to IgE [26]–[28]. Purified B cells from different individuals undergo class switching at low and variable frequencies and show considerable variation in chromatin changes associated with this process [23]. This variability has previously made these events difficult to analyse in the human system. Here we have taken advantage of the availability of both cultured cells from a human B cell line (CL-01) and purified tonsil B cells from several donors. Although the CL-01 cell line was initially reported to undergo CSR to IgG, IgA and IgE following cytokine and CD40 stimulation [27], [29] several laboratories, including our own, have found that this line now appears to have lost this ability (E. Max personal communication). While this would be a limitation for the analysis of the combined steps of immunoglobulin class switching (germline gene transcription, DNA recombination and B cell differentiation into immunoglobulin-secreting plasma cells), it reduces complexity to the single, essential, initial step of germline gene transcription: an event that occurs in the CL-01 cells and takes place in all primary human B cells, rather than a minor population [28]. This combination of sample materials allows us to robustly identify key chromatin remodelling events that occur at the Ig locus upon stimulation of εgermline transcription (εGLT) in the human system.

## Results

### Effect of IL-4 stimulation on CL-01 cells

**Figure 1:**
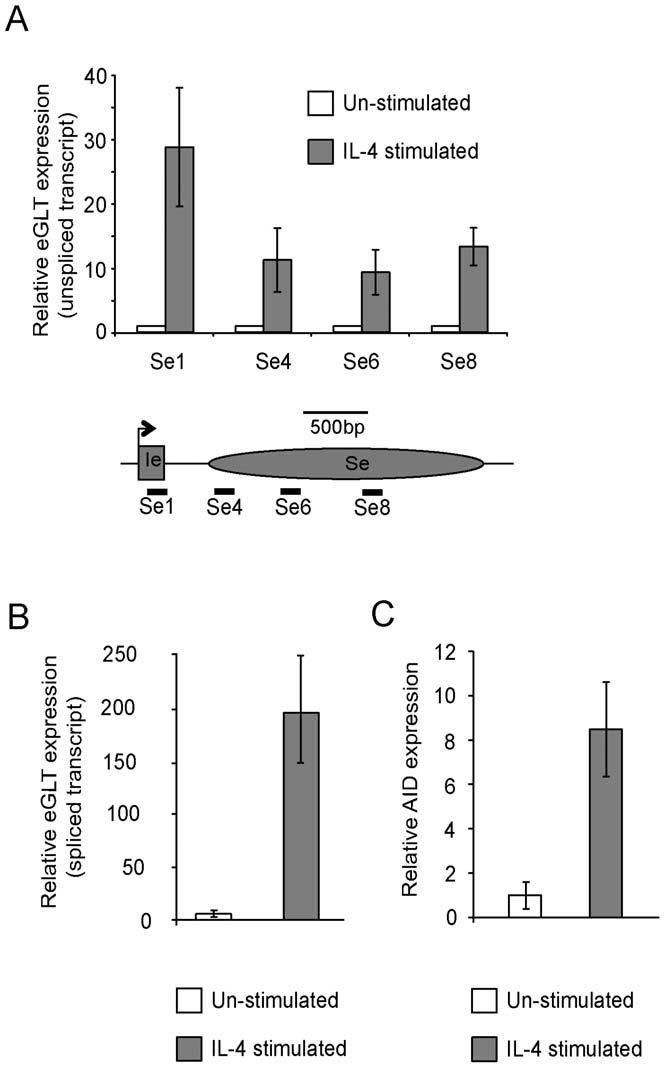
Analysis of ε germline gene transcripts and AID expression in CL-01 cells. Steady state transcript levels were quantified by qRT-PCR using HPRT as an endogenous control. All results are represented as changes relative to unstimulated CL-01 cells. The mean result from 3 separate experiments is shown. Error bars display standard deviation. Induction of (A) primary (un-spliced) ε germline gene transcripts, (B) mature (spliced) ε germline transcripts and (C) AID, in CL-01 cells following IL-4 stimulation for 72 hours. A schematic representation of the Ig ε locus is shown in panel A, with the elements approximately to scale, indicating the location of the primer/TaqMan probe sets used for the analysis of primary ε germline gene transcripts. Mature (spliced) ε germline transcripts were detected using a forward primer and TaqMan probe located in Iε and reverse primer in C_H_ ε exon 1 (not shown).

We began by using the CL-01 cell line to investigate the chromatin changes associated with the initiation of CSR to IgE in human B cells. These cells have been reported to initiate germline transcription of this region in response to IL-4 [27], [29], [34]. In order to investigate the human IgE switch region at high resolution, PCR primers were designed at unique sites spanning a region from Iε-Sε (Fig. S1). Four primers sets spanning Iε-Sε were used to quantify the expression level of primary (unspliced) ε germline transcripts (Fig. 1A). As expected, unstimulated cells expressed low levels of εGLT. Consistent with changes associated with the early stages of CSR, addition of IL-4 to the cultures for 72 hours resulted in a 10- to 30-fold increase in the primary transcript expression level. At this time point the change in the level of spliced εGLT was even greater, over 150 fold (Fig. 1B), while AID levels increased 8 fold (Fig. 1C), compared to the unstimulated cells.

**Figure 2:**
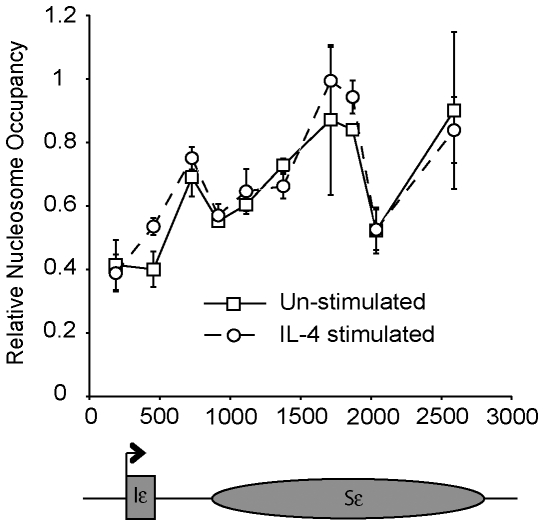
Nucleosome occupancy over Sε in CL-01 cells. Nucleosome occupancy over Sε was assessed in a mononucleosome chromatin fraction and compared to genomic DNA. The relative abundance of each primer location following ChIP was determined by qPCR. The mean result from 3 separate chromatin preparations is shown. Error bars display standard deviation. Data from un-stimulated cells is shown by squares and solid lines and stimulated cells by circles and dashed lines. A schematic representation of the Ig ε locus is shown, with the elements approximately to scale, indicating the position of each primer/TaqMan probe set plotted on the X axis.

Given the marked effect of IL-4 on transcription, we asked whether the addition of IL-4 also altered chromatin structure and acquisition of histone modifications over this region. The average nucleosome density (nucleosome occupancy) across Iε-Sε (Fig. 2) was determined, as previously described [31], to investigate the gross chromatin structure and thus general accessibility of the IgE switch region. Although nucleosome occupancy did not change following IL-4 treatment, nucleosome density increased from Iε towards the 3′ end of Sε in both the stimulated and un-stimulated cells. The presence of the histone variant H2A.Z is known to correlate with transcriptionally accessible chromatin structures [35], thus the accumulation of this variant was also investigated. Consistent with the nucleosome occupancy data, no changes in H2A.Z levels were seen upon IL-4 stimulation (Fig. S2).

**Figure 3:**
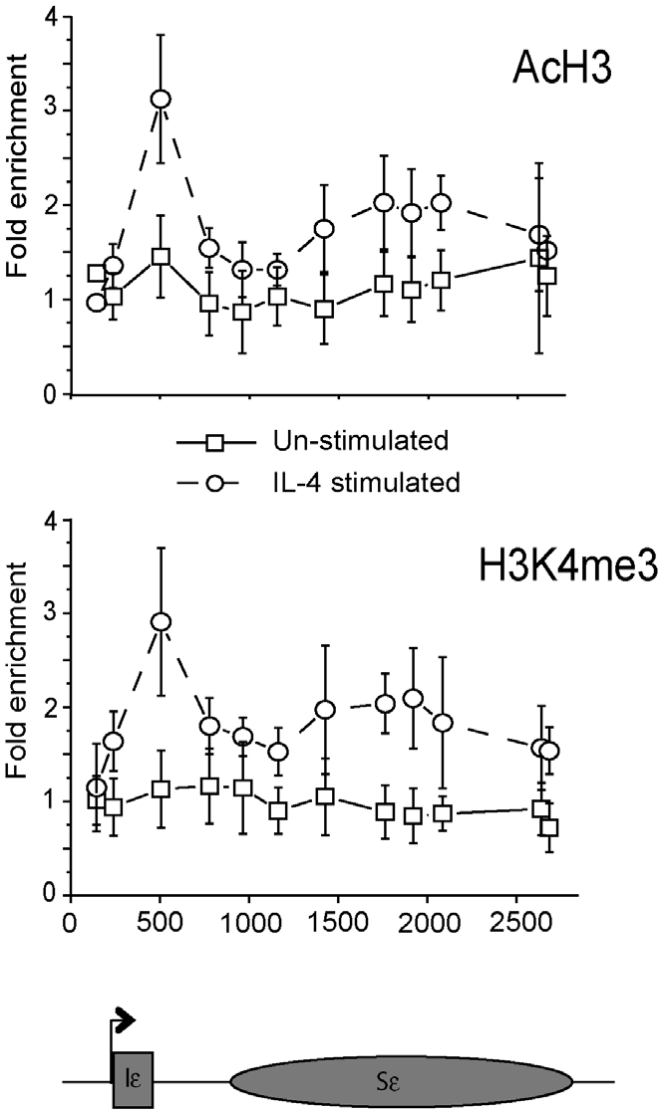
ChIP analysis of histone modifications at the Ig ε locus in CL-01 cells following IL-4 stimulation. Levels of di-acetylated (K9 and K14) histone H3 (top panel) and histone H3 tri-methylated at K4 (bottom panel) were assessed by ChIP using size-selected native chromatin to ensure high resolution. Analysis of histone modifications was carried out in CL-01 cells cultured for 72 hours with or without IL-4 stimulation. Mean results from 3 separate chromatin extractions are plotted as fold enrichments over an input control. Data from unstimulated cells is shown by squares and solid lines and stimulated cells by circles and dashed lines. Error bars show standard deviations. A schematic representation of the Ig ε locus is shown below the graphs, with the elements approximately to scale, indicating the position of each primer/TaqMan probe set plotted on the X axis.

We next surveyed the switch region for a range of histone modifications commonly associated with gene activation or repression. Following IL-4 stimulation, there were significant increases in the diacetylation of lysines 9 and 14 on histone H3 (AcH3) and tri-methylation of histone H3 lysine 4 (H3K4me3, Fig. 3). Although both of these modifications were increased across the whole Iε-Sε region, they were particularly enriched near the DNA encoding the Iε exon 5′ splice donor site and were markedly less abundant 3′ of Iε and at the 5′ end of Sε. IL-4 stimulation resulted in no significant change in histone H4 acetylation (AcH4) or histone H3K4 di-methylation (H3K4me2, Fig. S3), although these modifications were enriched in the vicinity of the Iε splice donor in both conditions. Little or no change was observed upon stimulation in H3 tri-methylation at K9 (H3K9me3), K27 (H3K27me3) or K36 (H3K36me3) (Fig. S3). However H3K9me3 and H3K27me3 levels were slightly depleted relative to input (values<1), indicating that these characteristic marks of inactive chromatin modifications are under-represented in this region.

### Effect of IL-4 and CD40 stimulation on CL-01 cells

Although IL-4 stimulation is sufficient to initiate εGLT expression (Figs. 1A and 1B), a “second signal” such as CD40 ligation, is required to initiate class switch recombination [10], [36]. IL-4 stimulation of CL-01 cells results in greatly increased tri-methylation of lysine 4 and acetylation of histone H3 around Iε (Fig. 3). We wished to determine whether the addition of the second signal altered the pattern of accumulation of these histone modifications over Sε. In addition to the increase in H3 acetylation and K4 tri-methylation (Fig. 3), previous studies had demonstrated increased tri-methylation of histone H3 lysine 9 at switch regions following stimulation of CSR [23] [25]; therefore these modifications were chosen for further analysis following culture of CL-01 cells with IL-4 and anti-CD40 antibody (Fig. S4).

No further changes in histone modifications were seen upon addition of anti-CD40 (and IL-4) to the cultures, compared to IL-4 alone. As for the IL-4 stimulated cells, AcH3 and H3K4me3 levels increased dramatically near the Iε exon 5′ splice site but were increased to a lesser extent over the switch region; H3K9me3 did not change in response to either mode of stimulation (Fig. S4).

### Effect of IL-4 and CD40 stimulation on primary human B cells

**Figure 4:**
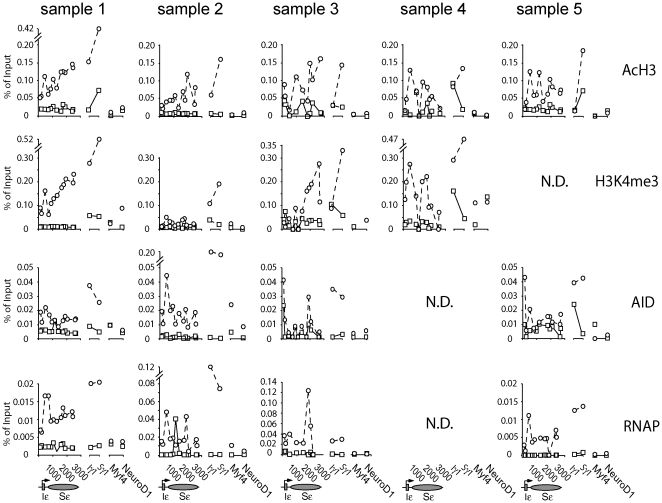
ChIP analysis of histone modifications at the Ig ε locus in primary human B cells. Levels of pan-acetylated histone H3, histone H3 tri-methylated at K4, RNA polymerase II (RNAP) and AID were assessed by ChIP using formaldehyde crosslinked chromatin from primary human B cells stimulated with IL-4 and anti-CD40 for 48 hours. Results from the individual donors for Sε primers sets are plotted alongside those for 5′Iγ1 and Sγ1 and two non-B cell expressed genes (*Myf4* and *NeuroD1*) for comparison. Data from unstimulated cells is shown by squares and solid lines and stimulated cells by circles and dashed lines. A schematic representation of the Ig ε locus is shown below the graphs, with the elements approximately to scale, indicating the position of each primer/TaqMan probe set. Additional (non-IgE) probes are also annotated on the X axis. N.D. – not determined.

To gain greater insights into the changes in Sε chromatin structure associated with human class switch recombination, we extended our studies to total primary B cells purified from tonsils from five human donors (Fig. 4). Cells were stimulated with IL-4 and anti-CD40 for 48 hours, when the highest levels of germline transcripts were observed in these cells (data not shown). In primary human B cells, stimulation with IL-4 and anti-CD40 results in CSR to IgG and IgE [28], [37], [38]. In order to investigate whether histone modification occurs differentially at IgG versus IgE, histone H3 acetylation and K4 tri-methylation were also measured at the Iγ1 promoter and a unique site within the γ1 switch region. Extensive sequence similarity within the IgG subtypes prevented a high resolution analysis of this gene. For comparison, histone modifications were also investigated at two genes not expressed in B cells: *myf4* (a transcription factor involved in myocyte differentiation) and *NeuroD1* (a transcription factor involved in neurogenesis).

Although specific distribution patterns were variable in the primary B cells, histone H3 acetylation (AcH3) levels increased (up to 20-fold) in all samples over Iε and Sε following stimulation with IL-4 and anti-CD40 (Fig. 4). As was seen in the CL-01 cells, in samples 1, 4 and 5, a distinct peak of acetylation was seen at the Iε exon 5′ splice site; no such peak was seen in sample 2 or 3, although acetylation levels did increase significantly following stimulation. With the exception of the Iε primers in sample 3, acetylation levels tended to be low 5′ of I exons but were significantly higher across the switch regions. In all samples except sample 3, acetylation levels were higher at the γ1 primer sets than the corresponding ε region. By comparison, H3 acetylation was low at the non-B cell genes.

Histone H3 lysine 4 tri-methylation levels across the ε locus were also increased upon stimulation of primary human B cells, in the three out of four donors (1, 3 and 4) for whom data were available (Fig. 4), again in agreement with the CL-01 data (Fig. 3). As observed for H3 acetylation, levels of stimulation were variable in the different donors. In two donors (1 and 4, to a greater or lesser extent, respectively) a peak of H3K4 tri-methylation is see at the Iε exon 5′ splice site (Fig. 4). As was also observed for H3 acetylation, H3K4 tri-methylation was higher at the 3′ end of Sε than was seen in the CL-01 cells. Elevation of H3K4me3 levels was also observed at the γ1 locus and was as much as two-fold higher than at the ε locus. Compared to the switch regions, levels of H3K4 tri-methylation were reduced at *Myf4* and *NeuroD1* in stimulated B cells.

We also profiled AID and RNA polymerase II (RNAP) binding patterns in primary B cells. In four individual experiments, we detected increased but variable RNAP and AID occupancy across the ε and γ1 loci upon stimulation (Fig. 4). As was true of the histone modifications, a trend towards a peak of AID and RNAP was seen at the Iε exon 5′ splice site in samples 1, 2 and 5. Interestingly, sample 3 showed little AID accumulation despite displaying robust H3 acetylation, H3K4 tri-methylation and RNAP accumulation. In stimulated B cells AID and RNAP levels were higher at γ1 than ε, while levels were lower at *Myf4* and *NeuroD1*.

To further investigate the chromatin structure of the IgE switch region prior to CSR, CpG DNA methylation across Sε was analysed by bisulphite modification in primary human B cells isolated from 3 donors (Fig. S5). Levels of DNA methylation were not significantly changed following IL-4 and anti-CD40 stimulation of B cells. Further, CpG sites located just upstream of the I exon 5′ splice site (site 176) exhibited reduced levels of methylation compared to surrounding regions while the sites at the 3′ end of Sε (sites 2081–2313) had slightly reduced levels of methylation than the 5′ end. Two further sites, one within Iε (98) and one at the 5′ end of Sε (580), also displayed noticeably lower levels of methylation than their surrounding sites.

## Discussion

In this study, we sought to identify changes in chromatin conformation within the human immunoglobulin heavy chain ε locus upon stimulation by treatments known to activate class switching to IgE. We initially took advantage of the CL-01 human B cell line to measure changes in histone modifications associated with the first stages of class switching in a highly reproducible system. These results were then used as reference points for the much more variable responses obtained with primary human B cells.

Measurements of nucleosome distribution across the ε switch region at high resolution (one primer/probe set every 220 bp on average) in CL-01 cells revealed that, although the density of nucleosomes (occupancy) generally increased towards the 3′ end of Sε this pattern of distribution was not affected by IL-4 stimulation (Fig. 2). Reduced nucleosome occupancy at transcription start sites is thought to be associated, in part, with increased levels of the histone variant H2A.Z [35]. However, we found that levels of H2A.Z were largely constant across Sε (Fig. S2), and not higher at the promoter, nor did they change significantly upon IL-4 stimulation.

The presence of nucleosomes in the switch region does not preclude AID attack; AID can readily deaminate deoxycytidine residues on transcriptionally active nucleosomal DNA *in vitro* [39]. However, the observed relative depletion of nucleosomes in the promoter region in unstimulated and IL-4 stimulated cells reveals that this region has increased accessibility to trans-acting factors (transcription factors, polymerase and AID) even before the activation of germline gene transcription. This conclusion is consistent with, and supports, our previous findings showing detectable levels of ε germline gene transcripts in resting B cells [28].

Cerutti and co-workers first made use of the CL-01 cell line to study human B cell activities, utilising CD40-ligand-expressing cells or an anti-CD40 mAb, to stimulate class switching to IgG and IgE. However, we have been unable to stimulate these cells to undergo class switching. This result confirms findings from other groups that, over time, these cells have lost their ability to undergo class switching. Despite their inability to undergo the later stages of recombination, we find that CL-01 cells nonetheless display characteristic remodelling of the chromatin at the IgE locus, including increases in histone H3 acetylation and tri-methylation of H3 at K4 (Fig. 3). The data demonstrate that these chromatin changes occur independently of recombination, a finding supported by the primary B cell data where chromatin changes in the bulk population are clearly visible despite a low frequency of cells switching to IgE.

That histone H3 acetylation and H3K4 tri-methylation was observed at the ε locus in both the CL-01 and primary B cells is not surprising; these chromatin marks generally correlate strongly with transcriptional activation [19], which is observed in every B cell upon IL-4 and CD40 stimulation [28]. Our current high resolution mapping of these histone modifications extends our previous findings [23] and is largely in agreement with several previous reports identifying changes in these histone modifications in response to CSR induction, although these were in all cases carried out at low resolution and in mice [20]–[22], [24], [25]. Tri-methylation of H3K4 is commonly detected proximal to transcription start sites in transcriptionally active genes [18], [40]. However, because H3K4me3 has been detected in human Sε (Figs. 3 and 4) and murine Sγ1 [24], as well as recombining V_H_ genes [41], [42], our data supports the hypothesis that H3K4me3 is also a mark of recombinationally active chromatin. Indeed, the V(D)J recombinase RAG2 contains an H3K4me3-binding PHD domain that is required for its proper function [43]–[45]. It is possible that a yet undiscovered PHD-containing cofactor is involved in the proper targeting of AID to H3K4me3-enriched activated switch regions.

We did not observe the significant changes in histone H4 acetylation or tri-methylation of histone H3 at lysine 9 within switch regions that had previously been reported in murine [21], [22], [25] and human B cells [23]. This demonstrates that analyses of histone modifications at single points over large loci can give misleading impressions of general levels of enrichments over these regions. Additionally, different constant regions could utilize different histone modifications to promote CSR. Previous studies have described switch recombination at γ loci, while we particularly noted H3K9me3 accumulation at Sμ and Sγ regions [23]. Our present high resolution analysis however has focused on Sε. Wang et al. [24] also observed induction of H3K36me3 just 3′ of murine Sγ1 in stimulated splenic B cells. We did not detect a corresponding increase in H3K36me3 levels at the 3′ end of Sε in CL-01 cells (Fig. S3). In addition to target identity and species-specific differences, the size of the respective switch regions could explain this difference. Because mouse Sγ1 is approximately 10 kb in size, the observed elevation in H3K36me3 could simply reflect its association with transcriptional elongation [18], [46]–[48], not a specific feature of switch region chromatin. Sε is approximately 2 kb in length and thus may be too close to the transcription start site to accumulate detectable H3K36 tri-methylation marks.

The histone modifications associated with silent heterochromatin, tri-methylated H3K27 and K9, are already depleted at Sε before stimulation. Similarly, marks of active chromatin, H4 acetylation and H3K4me2 are elevated in this region before stimulation. None of these modifications are significantly affected by IL-4 stimulation, but provide an environment permissive for AID deposition and action. These results support a model in which the chromatin structure is “poised” within the switch regions prior to cytokine stimulation [28].

In our primary human B cell studies we observed quantitative differences in the enrichment of acetylated H3 and H3K4me3 between activated Sε and Sγ1 (Fig. 4). Broadly, the elevated levels of these histone modifications at Sγ1 compared to Sε correlated with greater RNAP occupancy and to a lesser extent AID distribution. While it would have been desirable to perform a high-resolution analysis of Sγ1, the high degree of sequence repetition at the IgG genes prevented this. Thus, we cannot discount the possibility that these differences do not reflect the “general” level of modification across the γ1 locus. However, at least in places, Sγ1 is more highly modified than anywhere within Sε. These findings suggest that transcriptional activity, and in turn AID accumulation, at the switch regions is likely to be facilitated by the extent of histone modification over the genes; this could explain the preferential switching to γ compared to ε C_H_ genes in response to IL-4 and CD40 signalling.

In both the CL-01 cells (Fig. 3) and primary human B cells (Fig. 4) the nucleosomes near the Iε exon 5′ splice site are extensively modified and this coincided with a localised reduction in DNA methylation and accumulation of RNAP and AID in human B cells. A growing body of literature reveals a link between chromatin structure and splicing [49]. Interestingly, Sims et al. [50] found that the H3K4me3-binding chromatin remodelling factor CHD1, which binds spliceosomal components, is required for efficient RNA splicing. These data may go some way toward explaining the interesting earlier findings that implicated the requirement for the GLT splice site for successful CSR [51] and supports the recent observation that AID associates with paused RNA polymerase through an interaction with the spliceosome factor spt5 [52]. It is possible that the altered chromatin structure over the splice site may not only recruit complexes that are essential for germline gene transcript processing, but also complexes responsible for the process of DNA cleavage and recombination.

The use of the CL-01 model system allowed us to obtain highly reproducible data for changes associated with IL-4/anti-CD40 stimulation, which included increased histone H3 acetylation and H3K4 tri-methylation. In the corresponding studies in primary B cells, the specific distribution patterns varied among donors, but comparison to the CL-01 data makes it clear that the overall trends in primary B cells are similar to those in CL-01 cells. In the five donors investigated, no correlation could be seen between the levels of histone modification at Sε at 48 hours and IgE secretion at 12 days. Cell death is significant in the long-term (12 day) cultures (Supplemental Table S1), yet this does not appear to be a limiting factor for IgE production. We suggest that while histone modification is necessary for CSR it is not sufficient, and other factors must be involved in determining the success of CSR in individual B cells.

Abnormal IgE production is associated with a range of pathologies, including asthma, allergic rhinitis, Hyper-IgE and Hyper-IgM Syndromes. The development of therapeutic approaches to controlling these disease states necessitates the elucidation of mechanisms underlying CSR to IgE in human systems. Our findings suggest that approaches that specifically target the chromatin structural state in the switch region could be employed, for example by manipulating the chromatin structure of Sε using methods that would not affect switching to other C_H_ genes [53].

## Materials and Methods

### Primary Human B Cell purification

Human B cells were isolated from tonsils collected from patients undergoing routine tonsillectomies at the Evelina Children's Hospital (Guy's and St. Thomas' NHS Foundation Trust - ethics approval from Guy's Research Ethics Committee). The patients were all aged between 2 and 14, had no history of asthma, any known allergies or long standing medical conditions (except tonsillitis) and were not taking any medications. The patients' parents or legal guardians gave informed written consent for participation in this study. Total B cells were isolated from the tonsil as previously described [30]. B cell purity was assessed by flow cytometry using fluorescently-labelled antibodies (DakoCytomation) and a FACSCalibur™ flow cytometer (BD Biosciences). B cell populations were routinely >95% CD19^+^, with <5% contaminating CD3^+^ T cells. Generally, around 60% of these cells expressed IgM, with <2% IgG or IgE expressing cells (data not shown).

### Cell culture

B cells were cultured in 24-well plates (Nunc) at 0.5×10^6^ cells/mL in RPMI medium (Invitrogen Ltd.), supplemented with transferrin (35 µg/mL, Sigma-Aldrich Company Ltd.), insulin (5 µg/mL, Sigma-Aldrich Company Ltd.), penicillin (100 IU/mL), streptomycin (100 µg/mL), glutamine (2 mM) (all Invitrogen Ltd.) and 10% foetal bovine serum (FBS) (Hyclone, Perbio Biosciences Ltd.). Where indicated, media were supplemented with 1 µg/mL anti-CD40 antibody (G28.5, ATCC) and 200 IU/mL of recombinant human IL-4 (R&D Systems Ltd.). Unless specified in the text, cells were cultured for 48 hours prior to extraction of chromatin (see below) and for 12 days for analysis of IgE production. At the 48 hour time point cell viability is routinely 80–90%, as judged by trypan blue exclusion. Cell viability following 12 days in culture, as judged by flow cytometry, was more variable and is displayed for each donor in Supplemental Table S1.

CL-01 cells were cultured in RPMI 1640 medium with Glutamax (Invitrogen Ltd.) supplemented with 5% FBS (ATCC) and antibiotics. Where indicated, cells were cultured with 200 IU/mL of recombinant human IL-4 (R&D Systems Ltd.) and 1 µg/mL anti-CD40 monoclonal antibody (mAb) (G28.5, ATCC). To determine whether CSR had taken place after 7 days stimulation with IL-4 and anti-CD40, cells were stained for extracellular IgG or IgE and analysed by flow cytometry and the secretion of soluble IgE and IgG was investigated by ELISA [28]. No surface bound or secreted IgE or IgG was detected from the CL-01 cells following stimulation (data not shown). The CL-01 cells were also stimulated with IL-4 in combination with trimeric CD40-ligand [27], however CSR to IgG and IgE could not be detected (data not shown).

### Detection of IgE

Secretion of IgE was analysed by ELISA as previously described [28]. Briefly, Maxisorp plates (Nalge Europe Ltd.) were coated with polyclonal mouse anti-human IgE (DakoCytomation) in sodium carbonate buffer (pH 9.8) overnight at 4°C. Unbound sites were blocked with 2% non-fat milk powder (Marvel) in PBS/0.05% Tween (Sigma-Aldrich Company Ltd.). Samples were added and the plates were incubated for 16 hours at 4°C; NIP-IgE (JW8/5/13, ECACC, UK) was used to construct a standard curve. IgE was detected by mouse anti-human IgE conjugated to HRP (DakoCytomation) diluted 1/1000 in PBS/Tween 20 0.05%/1% non-fat milk powder for 4 hours at room temperature and revealed with OPD (Sigma-Aldrich Company Ltd.), with a minimum detection limit of 2 ng/mL. Surface IgE was detected by flow cytometry using a goat anti-human IgE antibody (Vector Laboratories Inc., Burlingame, USA). The percentage of IgE^+^ cells and amount of IgE secreted for each sample are shown in Supplemental Table S1.

### RNA extraction and quantitative RT-PCR (qRT-PCR)

Total RNA was extracted from 2×10^6^ cells using the RNeasy RNA isolation kit (Qiagen). Genomic DNA contamination was removed from 10 µg of total RNA using the Turbo DNA-free Kit (Ambion). cDNA was generated using the SuperScript III First-Strand Synthesis SuperMix for qRT-PCR (Invitrogen Ltd.). qRT-PCR was performed using the ABI-7900HT machine and TaqMan Universal PCR Mastermix (Applied Biosystems). Relative transcript levels were determined by comparing Ct values from equivalent amounts of cDNA derived from untreated cells to those from other experimental samples, all normalized to the endogenous reference gene human HPRT (Applied Biosystems) (ΔΔCt analysis). Supplemental Table S2 and S3 list oligonucleotides used in qRT-PCR experiments.

### Nucleosome Occupancy Analysis

Mono-, di- and tri-nucleosome fractions of native chromatin were prepared and DNA extracted as previously described [31]. Genomic DNA was isolated using the DNeasy Blood & Tissue Kit (Qiagen) and used as a reference for quantitative PCR (qPCR). DNA samples were quantified using the Quant-iT PicoGreen dsDNA reagent (Invitrogen Ltd.). 2 ng of DNA were subjected to qPCR using multiple primer/probe sets and qPCR was performed as described for qRT-PCR. Relative nucleosome abundance was calculated using the formula: 2^Ct(genomic)-Ct(nucleosomal)^.

### Native *chromatin immunoprecipitation*

Native (non-formaldehyde-crosslinked) *chromatin immunoprecipitations* (ChIPs) were performed and analysed in the IL-4 stimulated CL-01 experiments as previously described [31]. Antibodies used in this study included: anti-acetyl H3 (Millipore, 06-599), anti-acetyl H4 (Millipore, 06-598), anti-H3K4me2 (Millipore, 07-030), anti-H3K4me3 (Millipore, CS200580), anti-H3K9me3 (Abcam, ab8898), anti-H3K27me3 (Millipore, 07-449), anti-H3K36me3 (Abcam, ab9050), anti-H2AZ (Millipore, 07-954), anti-RNA Polymerase II (Covance, 8WG16) and anti-AID (Abcam, ab5197).

### Formaldehyde crosslinked *chromatin immunoprecipitation*

All ChIP experiments analyzing chromatin from cells cultured with IL-4 and anti-CD40 mAb stimulation (CL-01 and primary human B cells) were performed by formaldehyde crosslinking, MNase-treatment and sonication of chromatin. Briefly, 1×10^8^ cells were fixed at 20°C for 4 minutes in 1% formaldehyde in 10 mL of culture medium. The cross-linking reaction was stopped by the addition of glycine to a final concentration of 125 mM. The cells were spun and the cell pellet resuspended in 1× PBS containing 125 mM glycine and incubated at 20°C for 5 minutes. Nuclei were isolated as detailed for primary human B cells [23] and CL-01 [31]. MNase digestion and chromatin extraction were performed as previously described [23]. Chromatin was sonicated to aid DNA fragmentation to a 100 bp–500 bp range. 10 µg of chromatin were diluted in 0.4 mL modified RIPA buffer (140 mM NaCl, 10 mM Tris pH 7.5, 1 mM EDTA, 0.5 mM EGTA, 1% Triton X-100, 0.01% SDS, 0.1% NaDeoxycholate) and incubated overnight at 4°C with 3–5 µg of the appropriate antibody and 25 µL of Protein G-magnetic beads (Active Motif) in the presence of protease inhibitor cocktail and 5 mM sodium butyrate (Sigma-Aldrich Company Ltd.). Beads were washed twice in IPWB1 (20 mM Tris pH 8.0, 50 mM NaCl, 2 mM EDTA, 1% Triton X-100, 0.1% SDS) and twice in IPWB2 (10 mM Tris pH 8.0, 150 mM NaCl, 1 mM EDTA, 1% NP-40, 250 mM LiCl, 1% Sodium Deoxycholate). Beads were resuspended in 100 µL of 10% Chelex 100 Resin (Bio-rad), boiled for 5 minutes, RNase-treated for 60 minutes at 37°C and proteinase K-treated for 30 minutes at 55°C. Samples were boiled for 10 minutes and DNA-containing supernatant was isolated for qPCR analysis. Equivalent volumes of isolated ChIP DNA and input DNA were subjected to qPCR. A standard curve was generated to convert the differences in Ct values to percent of input.

### qPCR analysis of ChIP experiments

Following extraction and purification of DNA from ChIPs, qPCR was performed to determine enrichment of target sequences. All primers used bound uniquely to the genome. Sε and negative control primer sets were designed using Primer Express (Applied Biosystems) and checked for unique alignment to the genome by BLAST analysis. Because of the repetitive nature of the human IgG genes a novel primer design strategy was employed to identify unique primer/probe pairs. Briefly, the γ1 switch region sequence was incrementally divided into short overlapping oligonucleotides of 18 to 25 bp in length. Oligonucleotides having a GC content of 40 to 60% and containing no repeats of greater than 4 nucleotides were selected for further analysis. UNAfold analysis was performed to exclude oligonucleotides that could form homo-dimers at 45°C or with annealing temperatures outside of the desired range (59 to 63°). Oligonucleotides that bound uniquely to Sγ1 were identified using FASTA. Finally, suitable oligonucleotide pairs were identified that produced amplicons of less than 190 bp in length and had a difference in annealing temperatures of less than 2°C. Primer pairs were checked for unique alignment by BLAST and probes designed using the Universal ProbeLibrary Assay Design Centre (Roche Applied Science). The location of all the IgE and IgG primer sets (relative to the I exon) is shown in Supplemental Table S3, and displayed graphically (Fig. S1). qPCR analysis was carried out as described above. Sufficient DNA was recovered from the native CL-01 cell histone ChIPs to allow accurate quantification of recovered samples using the Quant-iT PicoGreen assay (Invitrogen Ltd.); fold enrichment values have therefore been displayed for these ChIPs in Figures 1, 3, S2 and S3. Insufficient DNA was recovered from crosslinked Polymerase and AID ChIPs to allow accurate quantification. For all crosslinked ChIPs, recovered DNA was quantified using a standard curve, generated from genomic DNA serial dilutions, and expressed as percent recovery compared to input.

### Bisulphite modification analysis

Genomic DNA was extracted from 1×10^7^ cells using a Wizard® genomic DNA extraction kit (Promega, Madison, USA). CpG methylation site mapping was performed by bisulphite modification (BSM) of DNA, adapted from Frommer et al. [32], followed by PCR amplification and sequencing. 10 µg of genomic DNA were digested with Kpn I (New England Biolabs UK Ltd.) and purified by phenol extraction, followed by ethanol precipitation. DNA was denatured by treatment with 0.2 M NaOH at 37°C for 15 minutes. To this, was added 30 µl of 10 mM hydroquinone and 520 µl of 3 M sodium bisulphite pH 5 (both freshly prepared; Sigma-Aldrich Company Ltd.). Samples were incubated in the dark for 16 hours at 50°C prior to salt removal using the Wizard DNA clean-up system (Promega). DNA was desulphonated in a final concentration of 0.3 M NaOH at room temperature for 15 minutes, neutralised by adding 1 volume of 6 M ammonium acetate, precipitated with 3 volumes of ethanol and resuspended in 20 µL TE buffer.

Primers for the amplification of BSM DNA were designed using MethPrimer [33]. All PCR reactions were carried out on 2 µl of BSM DNA in a 50 µl reaction volume containing 1.5 mM MgCl_2_ and Hotstart Platinum Taq (Invitrogen Ltd.). PCR-amplified products were cloned using the TOPO® cloning system (Invitrogen Ltd.) and individual colonies sequenced. 20 sequences were collected for each CpG site. The following primer pairs were used for the amplification of BSM modified DNA: E1F TTTGTTGATTGGGATTATTAAGTT A, E1R CAAACAACCTCTCCCTCACAACTAC; E2F TTTTTTTTGTATGGGGA TATAGGAA, E2R CCCAACTCAAACCTAACTCAACTAA; E3F AGTTGAATTA GGTTGATTTGGATTT, E3R AACCTACTCACTCCAACTTTTAACC; E4F TGG GTTGAGTTGAGTTAGGTTAAAT, E4R CCCCTTACAAACAACAAACTCTTA T.

## Supporting Information

Table S1 **IgE production and cell viability of B cell cultures.** IgE production and cell viability of each primary human B cell culture was determined following 12 days stimulation with IL-4 and anti-CD40. Secreted IgE was determined by ELISA, the % of IgE^+^ cells and cell viability were determined by flow cytometry. UD. – Undetected.
(DOCX)

Table S2 **qRT PCR Assays.** Details of the assays used for quantitative RT-PCR analyses are given; AID and HPRT were detected by proprietary assays from Applied Biosystems. εGLT assays were designed “in-house” and used MGB dual labelled probes (Applied Biosystems).
(DOCX)

Table S3 **qPCR assays.** Details of the assays used for quantitative PCR analysis of ChIPs and unspliced (primary) εGLT assays are given; Sε assays were designed “in-house” and used dual labelled probes, γ1, *NeuroD1* and *Myf4* assays were designed to use dual labelled Universal Probe Library probes (Roche). The approximate genomic location of each assay is given.
(PDF)

Figure S1 **Location of qPCR primer sets across the IgE locus.** The location of the IgE qPCR primer sets is displayed on a graphical represention (to scale) of the IgE locus.
(TIF)

Figure S2 **ChIP analysis of H2A.Z deposition at the Ig ε locus in CL-01 cells.** H2A.Z deposition over Sε was investigated in CL-01 by ChIP using size-selected native chromatin. Cells were harvested following 72 hours culture with or without IL-4. Mean results from 3 separate chromatin extractions are plotted as fold enrichments over an input control. Data from unstimulated cells is shown by open squares and solid lines, stimulated cells are open circles and dashed lines. Error bars show standard deviations. A schematic representation of the Ig ε locus, with the elements approximately to scale, is shown below the graph indicating the position of each primer/TaqMan probe set plotted on the X axis.
(TIF)

Figure S3 **ChIP analysis of histone modifications at the Ig ε locus in CL-01 cells following IL-4 stimulation.** Histone modification over Sε was investigated in CL-01 by ChIP using size-selected native chromatin. Cells were harvested following 72 hours culture with or without IL-4. Mean results from 3 separate chromatin extractions are plotted as fold enrichments over an input control. Data from unstimulated cells is shown by open squares and solid lines, stimulated cells are open circles and dashed lines. Error bars show standard deviations. A schematic representation of the Ig ε locus, with the elements approximately to scale, is shown below each graph indicating the position of each primer/TaqMan probe set plotted on the X axis. The following histone modifications are shown: AcH4, di-methyl H3K4, tri-methyl H3K36, tri-methyl H3K9 and tri-methyl H3K27.
(TIF)

Figure S4 **ChIP analysis of histone modifications at the Ig ε locus in CL-01 cells following IL-4 and anti-CD40 stimulation.** Histone modification over Sε was investigated in CL-01 by ChIP using size-selected native (non-crosslinked) chromatin. Cells were harvested following 72 hours culture with or without IL-4 and anti-CD40. Mean results from 3 separate chromatin extractions are plotted as fold enrichments over an input control. Data from unstimulated cells is shown by open squares and solid lines; stimulated cells are open circles and dashed lines. Error bars show standard deviations. A schematic representation of the Ig ε locus, with the elements approximately to scale, is shown below each graph indicating the position of each primer/TaqMan probe set plotted on the X axis.
(TIF)

Figure S5 **Analysis of DNA CpG methylation over Sε and Sγ1.** CpG methylation was analysed across Sε by bisulphite modification of DNA followed by sequencing. Genomic DNA was extracted from tonsil B cells isolated from three donors, 20 sequences were collected for each CpG site. The percentage of methylated deoxcytidines found across the three donors is plotted; error bars show the standard deviation in the data between the three donors. The location of the CpG site is shown on the x axis: Numbers refer to the distance (bp) from the Iε start site and the location is displayed on the graphical representation of the IgE locus below; long vertical lines show each CpGs analysed, short lines show the location of CpGs that could not be analysed (distance from Iε is given below the graphic). The locations of 5 sites that have especially low levels of methylation are emphasised.
(TIF)
