# Supplementary material for: High Resolution Analysis of the Chromatin Landscape of the IgE Switch Region in Human B Cells
Source: PLoS One. 2011 Sep 20;6(9):e24571. doi: 10.1371/journal.pone.0024571 (PMC3176761; doi:10.1371/journal.pone.0024571)
Supplement: Table S1 — IgE production and cell viability of B cell cultures. IgE production and cell viability of each primary human B cell culture was determined following 12 days stimulation with IL-4 and anti-CD40. Secreted IgE was determined by ELISA, the % of IgE+ cells and cell viability were determined by flow cytometry. UD. – Undetected. (DOCX) [file pone.0024571.s001.docx]

| Donor | Secreted IgE  ng/mL | % IgE+ | % Cell viability |
| --- | --- | --- | --- |
| 1 | 40 | UD. | 10 |
| 2 | UD. | UD. | 30 |
| 3 | 38 | 0.34 | 20 |
| 4 | 2743 | 30 | 11 |
| 5 | 2 | 0.1 | 35 |

**Table S1**
