# Supplementary material for: High Resolution Analysis of the Chromatin Landscape of the IgE Switch Region in Human B Cells
Source: PLoS One. 2011 Sep 20;6(9):e24571. doi: 10.1371/journal.pone.0024571 (PMC3176761; doi:10.1371/journal.pone.0024571)
Supplement: Table S2 — qRT PCR Assays. Details of the assays used for quantitative RT-PCR analyses are given; AID and HPRT were detected by proprietary assays from Applied Biosystems. εGLT assays were designed “in-house” and used MGB dual labelled probes (Applied Biosystems). (DOCX) [file pone.0024571.s002.docx]

| Target Gene/location | Assay name | Oligo sequence/assay details |
| --- | --- | --- |
| AID | hAICDA RT-PCR assay | Applied Biosystems Gene Expression Assay (Part No. 433182) |
| HPRT | hHPRT RT-PCR assay | Applied Biosystems Gene Expression Assay (Part No. 4310890E) |
| IgE | Spliced εGLT RT-PCR assay- | F: CTGTCCAGGAACCCGACAGA  R: TGAAGCGCCGGGTCGTC  Probe: AGGCACCAAATG |

**Table S2**
