# Supplementary material for: High Resolution Analysis of the Chromatin Landscape of the IgE Switch Region in Human B Cells
Source: PLoS One. 2011 Sep 20;6(9):e24571. doi: 10.1371/journal.pone.0024571 (PMC3176761; doi:10.1371/journal.pone.0024571)
Supplement: Table S3 — qPCR assays. Details of the assays used for quantitative PCR analysis of ChIPs and unspliced (primary) εGLT assays are given; Sε assays were designed “in-house” and used dual labelled probes, γ1, NeuroD1 and Myf4 assays were designed to use dual labelled Universal Probe Library probes (Roche). The approximate genomic location of each assay is given. (PDF) [file pone.0024571.s003.pdf]

Target Gene/location	Assay name	Oligo sequence/assay details
150bp 5' of Iε	5'Ie	F: CTCGGTGTGCATCGGTAGTG<br>R: CTCTGTTCTTGGGAAGTCGATTG<br>Probe: CAGCCTCACCTGACCCCCGC
Start of Iε	Ie	F:CACCCCATTTTTAGCTGAAAGC<br>R:CAGCTCCCAGATGATCAGTAACC<br>Probe:CTGAGGCAGAGCTCCCCCTACCCA
250bp 3' of Iε	Se1	F:ACAGCATCAACCAAGCTTCTTG<br>R:GCATTAGGCTGTTTCTAGCTTTCC<br>Probe:TCACTGCGTGGACTCTAGGCACTCCC
500bp 3' of Iε	Se2	F:ACTGGCTGATCTTGGCAAGTCC<br>R: TCGCCTCAGCTCAGTCTGTTAG<br>Probe: CTTGGCTGAGTGGACTAGACTGAGACGGG
750 bp 5' of Iε	Se3	F:AGGCGGGTTTAGCTGAGTTG<br>R: CTAGTCCACCTTAGGGCTTAGGATAAC<br>Probe:TGAGTGGACTGGGTAGAGGGAAATGAGCT
1Kb 5' of Iε<br>(start of Sε)	Se4	F:TGACCTGGACTTGGTTGAAATAAG<br>R:CACCCATTTTATCCTAAGTAGAGAACTG<br>Probe: TGGGCCGACACAGGAGTAGGGACA
1.2Kb 5' of Iε	Se5	F:TGACGACGGACAGGGTTAAAAG<br>R:TCATCCCAGCTAAATTCAGATCAC<br>Probe:TGGAGTGAGCAGGCCTTAAATTGGGC
1.4kb 5' of Iε	Se6	F:GGGTCAGGTTGAGGTTAACTGAAC<br>R:CCATCCACCTCAGTCCAGTTG<br>Probe:CTGGGCCTAAACTGGGTTTGGCTGG
1.65Kb 5' Iε<br>(Middle Sε)	Se7	F:GCCTGAGCTGTGATTGGAAGAC<br>R:AACCAGCTTAGTCAGCCCAACTAG<br>Probe:TGAGCTGGACAGACCTGAGCCAAGC
1.8Kb 5' of Iε	Se8	F:CTTGGATTATTGAACCGAATTGG<br>R:CTAGGTTCAGCTCTGCTCAGTTCAG<br>Probe:TGATTTAAACTGAGTTCTGCTGGGATAAGCTGATC
2.35Kb 5' of Iε<br>End Sε	Se9	F:GGTGGCTACTTTAGGTCAGCTTTG<br>R:TTAGCCAGGTGAGAATGGTCAGT<br>Probe:TGAGCTAAACTGGACCGGGCTAAATTGATC
2.4Kb 5' of Iε	Se10	F:GGCTAAGAGGAGCTGAGTCAGAAG<br>R: CCCTTGCAGGCAGCAAAC<br>Probe:AAGCTGGTTGAGCTGGCTGGACTGAA

50bp 5' Ig1	Ig1	F: GACGGCAGGGGCGGG<br>R: GTCTCAGCCCTTCCTGTTC<br>Probe: Roche Universal Probe Library (UPL) #44
1.2Kb 5' Ig1	Sg1	F: GAGCATCACAACGTCAGCAT<br>R: CAGATCTGCCCTGGCTCT<br>Probe :Roche Universal Probe Library (UPL) #10
Exon2 of NeuroD1	NeuroD1	F: GATCAAAAGCCCAAGAGACG<br>R: GCCTTCATGCGTCTCAATTT<br>Probe: Roche Universal Probe Library (UPL) #1
Exon1 of Myf4	Myf4	F:CGCTTCTATGATGGGGAAAAC<br>R: GCTCGTAGCCTGGTGGTTC<br>Probe: Roche Universal Probe Library (UPL) #10

Table S3
