# Supplementary figures and images for: High Resolution Analysis of the Chromatin Landscape of the IgE Switch Region in Human B Cells
Source: PLoS One. 2011 Sep 20;6(9):e24571. doi: 10.1371/journal.pone.0024571 (PMC3176761; doi:10.1371/journal.pone.0024571)

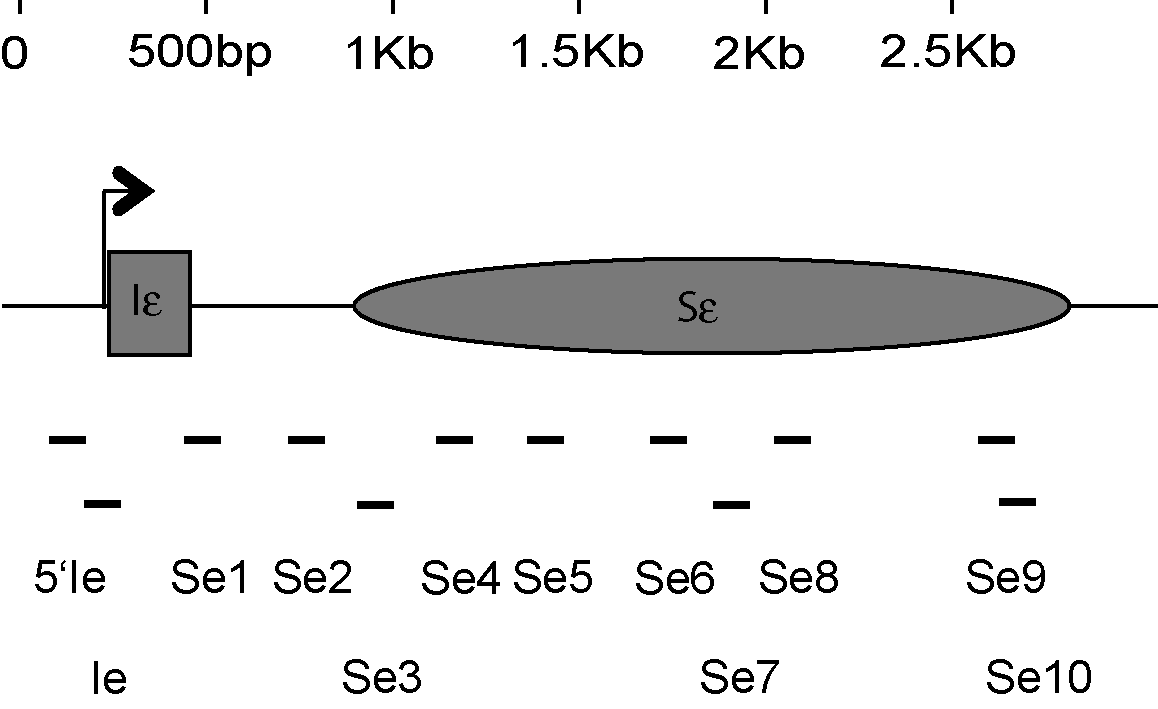

Supplement: Figure S1 — Location of qPCR primer sets across the IgE locus. The location of the IgE qPCR primer sets is displayed on a graphical represention (to scale) of the IgE locus. (TIF) [file pone.0024571.s004.tif]

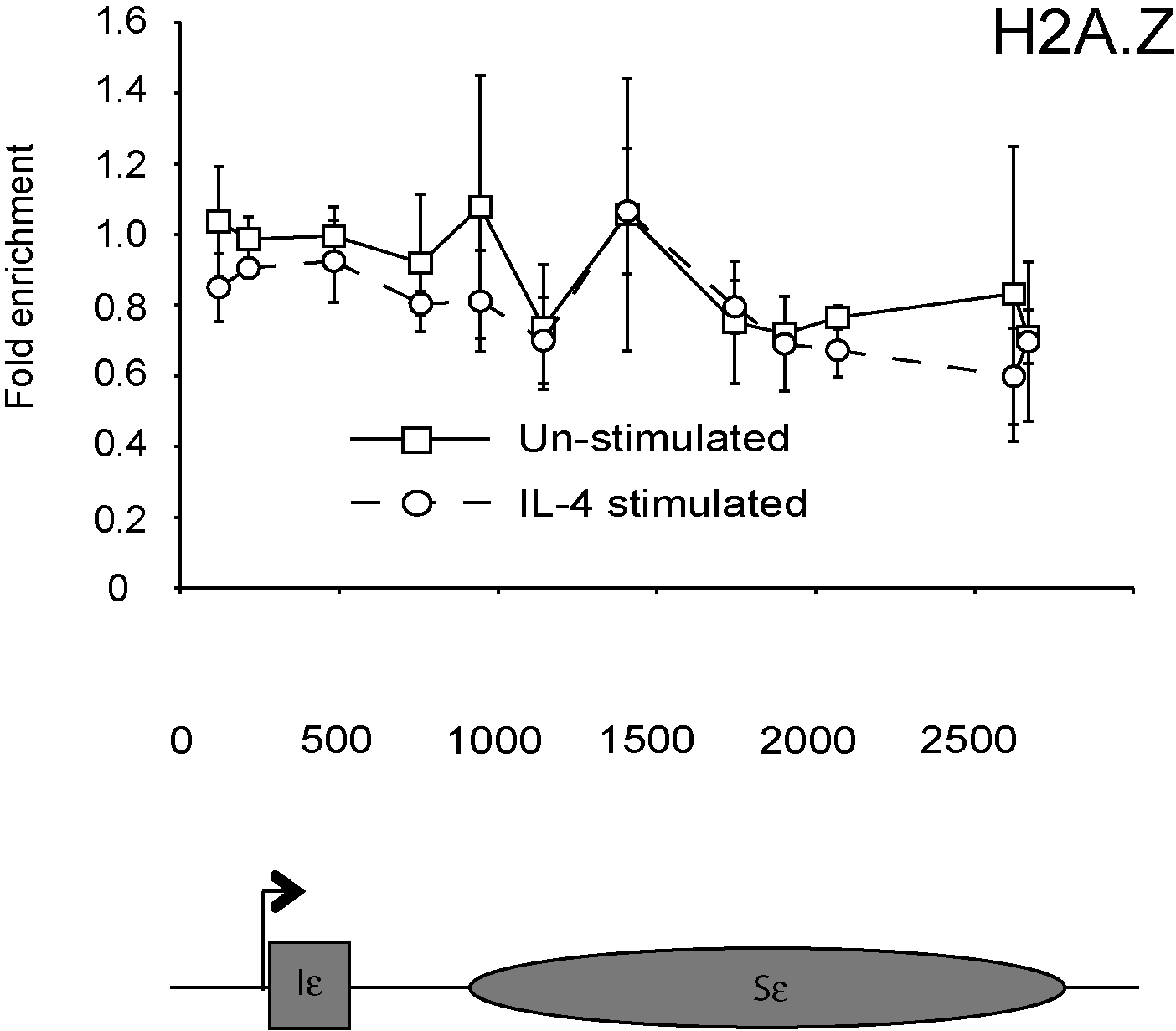

Supplement: Figure S2 — ChIP analysis of H2A.Z deposition at the Ig ε locus in CL-01 cells. H2A.Z deposition over Sε was investigated in CL-01 by ChIP using size-selected native chromatin. Cells were harvested following 72 hours culture with or without IL-4. Mean results from 3 separate chromatin extractions are plotted as fold enrichments over an input control. Data from unstimulated cells is shown by open squares and solid lines, stimulated cells are open circles and dashed lines. Error bars show standard deviations. A schematic representation of the Ig ε locus, with the elements approximately to scale, is shown below the graph indicating the position of each primer/TaqMan probe set plotted on the X axis. (TIF) [file pone.0024571.s005.tif]

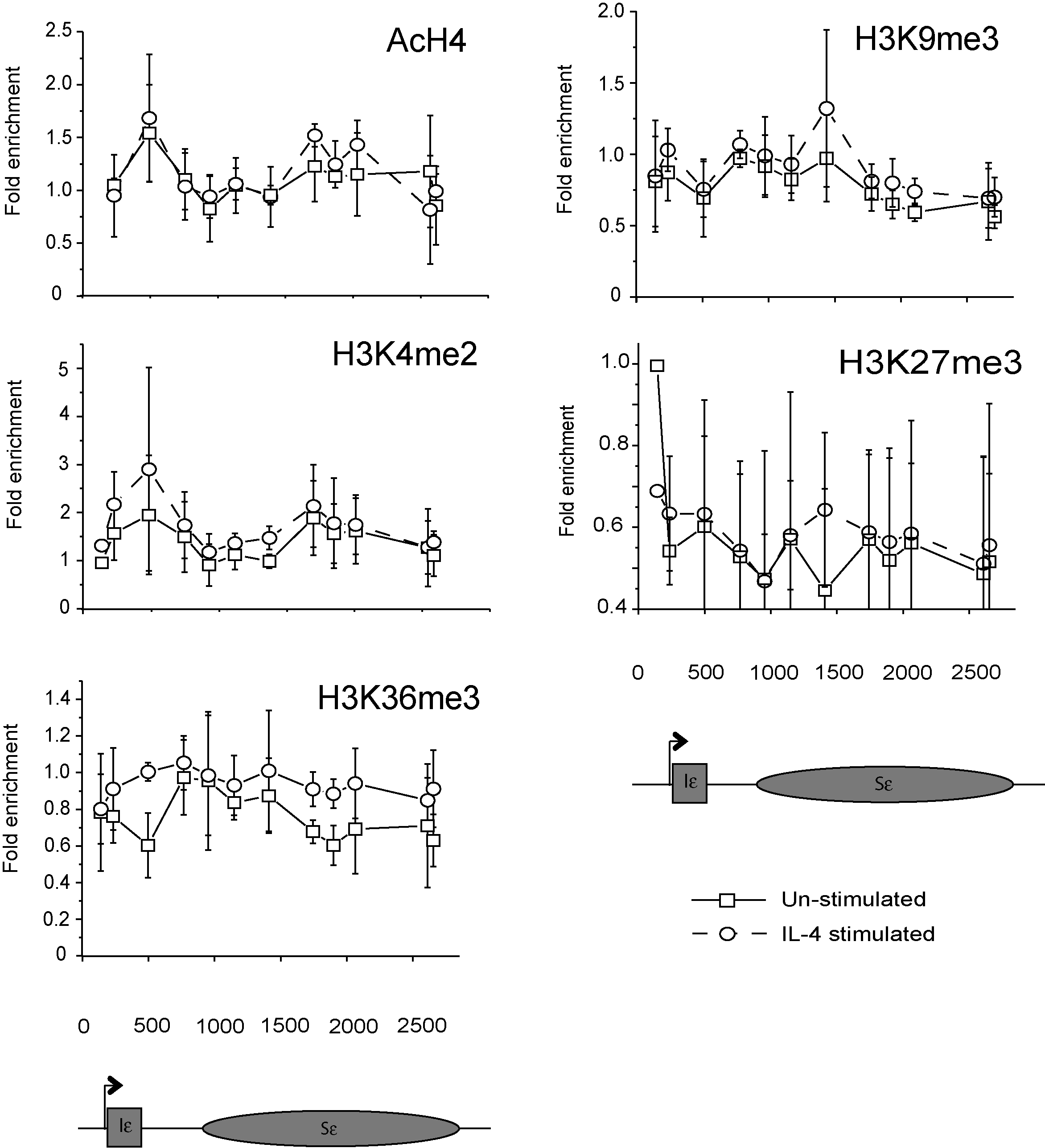

Supplement: Figure S3 — ChIP analysis of histone modifications at the Ig ε locus in CL-01 cells following IL-4 stimulation. Histone modification over Sε was investigated in CL-01 by ChIP using size-selected native chromatin. Cells were harvested following 72 hours culture with or without IL-4. Mean results from 3 separate chromatin extractions are plotted as fold enrichments over an input control. Data from unstimulated cells is shown by open squares and solid lines, stimulated cells are open circles and dashed lines. Error bars show standard deviations. A schematic representation of the Ig ε locus, with the elements approximately to scale, is shown below each graph indicating the position of each primer/TaqMan probe set plotted on the X axis. The following histone modifications are shown: AcH4, di-methyl H3K4, tri-methyl H3K36, tri-methyl H3K9 and tri-methyl H3K27. (TIF) [file pone.0024571.s006.tif]

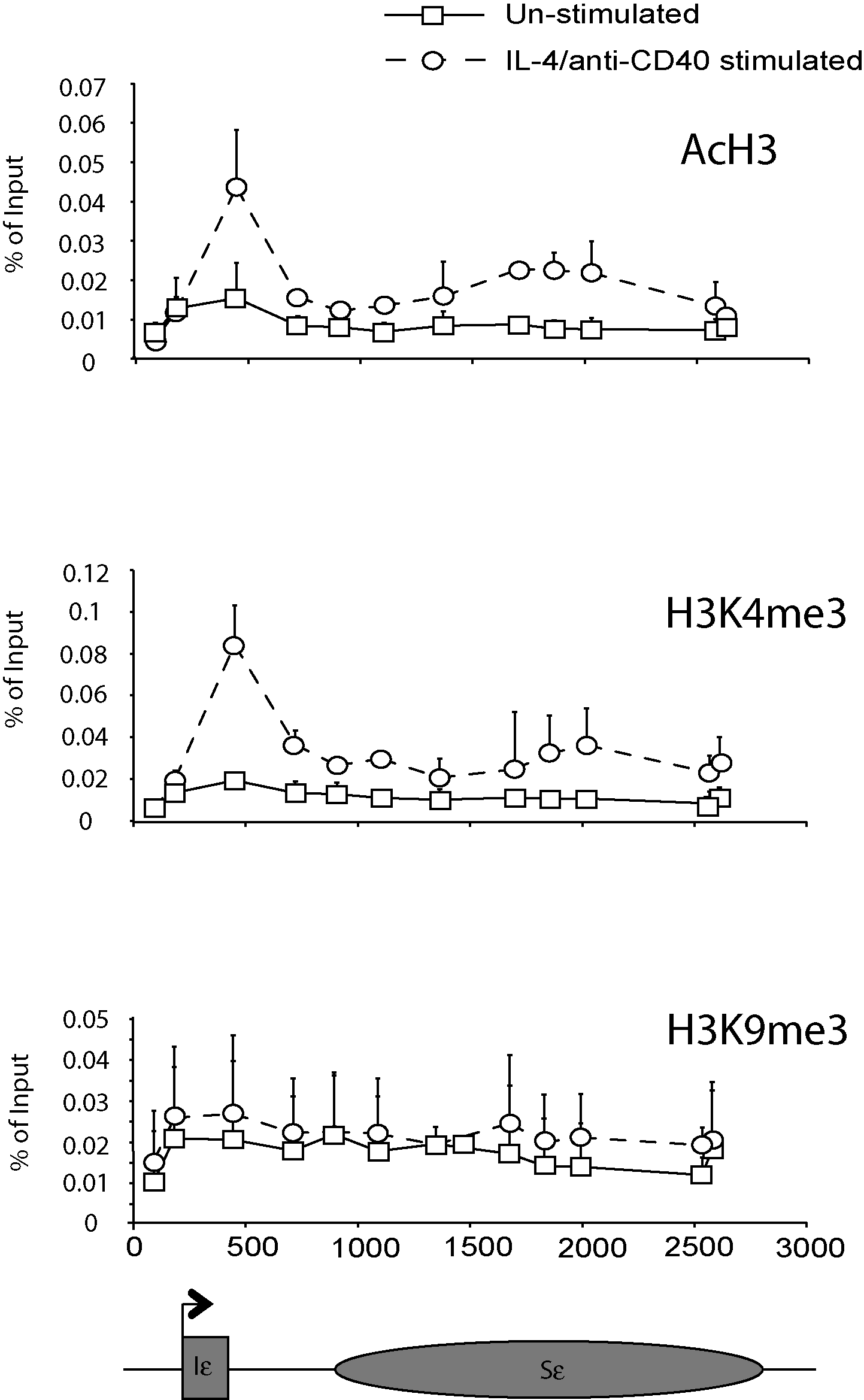

Supplement: Figure S4 — ChIP analysis of histone modifications at the Ig ε locus in CL-01 cells following IL-4 and anti-CD40 stimulation. Histone modification over Sε was investigated in CL-01 by ChIP using size-selected native (non-crosslinked) chromatin. Cells were harvested following 72 hours culture with or without IL-4 and anti-CD40. Mean results from 3 separate chromatin extractions are plotted as fold enrichments over an input control. Data from unstimulated cells is shown by open squares and solid lines; stimulated cells are open circles and dashed lines. Error bars show standard deviations. A schematic representation of the Ig ε locus, with the elements approximately to scale, is shown below each graph indicating the position of each primer/TaqMan probe set plotted on the X axis. (TIF) [file pone.0024571.s007.tif]

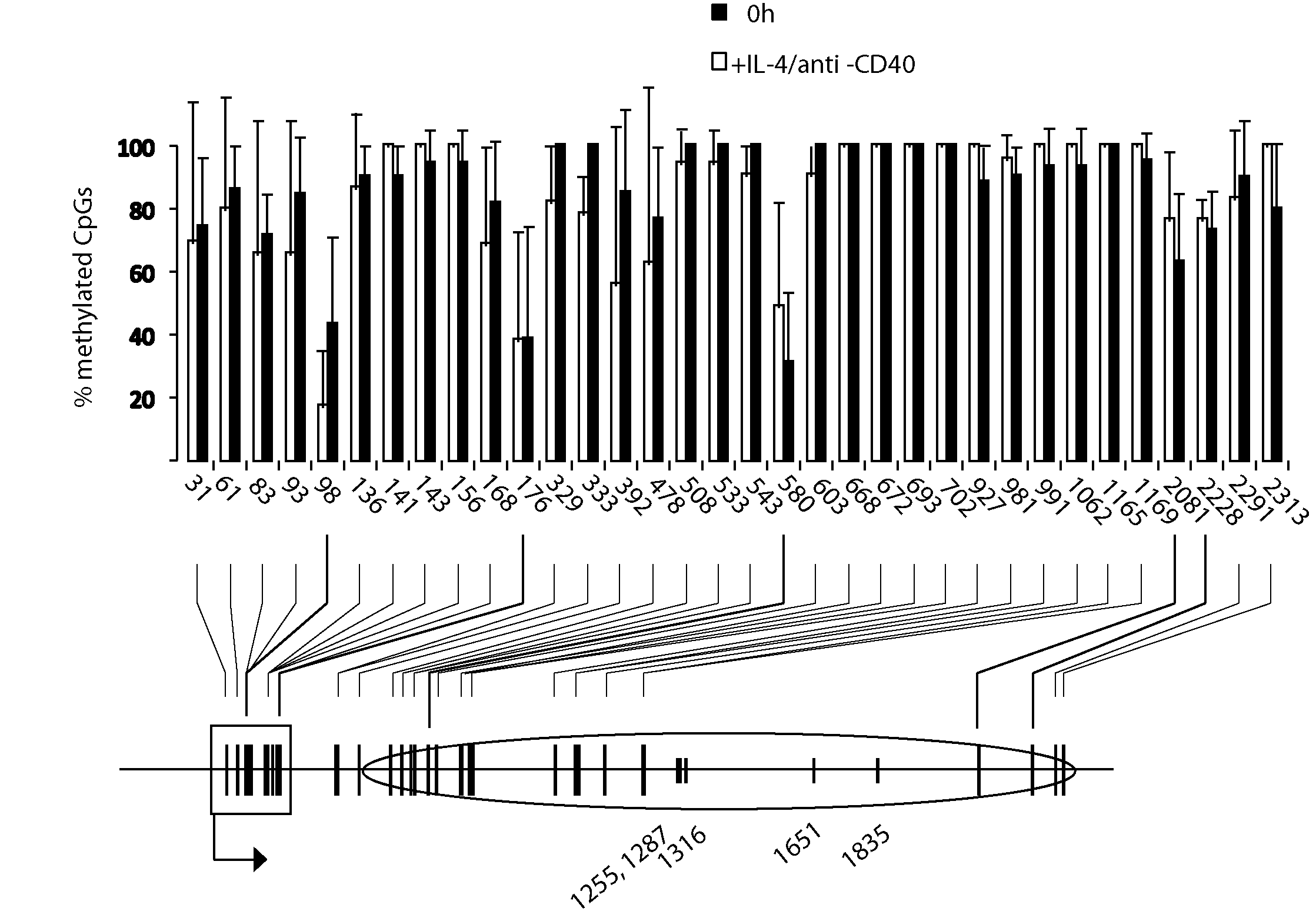

Supplement: Figure S5 — Analysis of DNA CpG methylation over Sε and Sγ1. CpG methylation was analysed across Sε by bisulphite modification of DNA followed by sequencing. Genomic DNA was extracted from tonsil B cells isolated from three donors, 20 sequences were collected for each CpG site. The percentage of methylated deoxcytidines found across the three donors is plotted; error bars show the standard deviation in the data between the three donors. The location of the CpG site is shown on the x axis: Numbers refer to the distance (bp) from the Iε start site and the location is displayed on the graphical representation of the IgE locus below; long vertical lines show each CpGs analysed, short lines show the location of CpGs that could not be analysed (distance from Iε is given below the graphic). The locations of 5 sites that have especially low levels of methylation are emphasised. (TIF) [file pone.0024571.s008.tif]
